# Supplementary material for: Approximating missing epidemiological data for cervical cancer through Footprinting: A case study in India
Source: eLife. 2023 May 25;12:e81752. doi: 10.7554/eLife.81752 (PMC10212556; doi:10.7554/eLife.81752)
Supplement: Figure 1—source data 1. [file elife-81752-fig1-data1.docx]

**Figure 1 – Source Data 1.** **Overview of availability of cervical cancer epidemiological data by state.**

| State/Group of states * | Sexual behaviour | Cervical cancer incidence ± | HPV prevalence † | Data availability level | CI5 registry ¤ | NCDIR registry ¤ |
| --- | --- | --- | --- | --- | --- | --- |
| Andhra Pradesh | X | X |  | Intermediate |  | *Hyderabad district* |
| Assam | X | X |  | Intermediate | Cachar, Kamrup Urban District | Cachar district, *Dibrugarh district*, Kamrup urban |
| Bihar | X |  |  | Low |  |  |
| Chhattisgarh | X |  |  | Low |  |  |
| Delhi | X | X |  | Intermediate |  | *Delhi* |
| Goa + Daman & Diu | X |  |  | Low |  |  |
| Gujarat + Dadra & Nagar Haveli | X | X |  | Intermediate | Ahmedabad | Ahmedabad urban |
| Haryana | X |  |  | Low |  |  |
| Himachal Pradesh | X |  |  | Low |  |  |
| Jammu & Kashmir | X |  |  | Low |  |  |
| Jharkhand | X |  |  | Low |  |  |
| Karnataka | X | X |  | Intermediate | Bangalore | Bangalore |
| Kerala + Lakshadweep | X | X |  | Intermediate | Kollam, Trivandrum | Kollam district, Thi'puram district |
| Madhya Pradesh | X | X |  | Intermediate | Bhopal | Bhopal |
| Maharashtra | X | X |  | Intermediate | Barshi & Paranda & Bhum, Mumbai, Poona, Wardha | *Aurangabad, Osamanabad & Beed*, Barshi rural, Mumbai, Pune, Wardha district, *Nagpur* |
| Manipur | X | X |  | Intermediate |  | *Manipur state, Imphal West district* |
| Orissa | X |  |  | Low |  |  |
| Other North Eastern States § | X | X |  | Intermediate | Mizoram, Tripura | Mizoram state, *Aizawl district*, Tripura state, *West Arunachal, Papumpare district, Meghalaya, East Khasi Hills district, Nagaland, Pasighat* |
| Punjab + Chandigarh | X | X |  | Intermediate |  | *Patiala district* |
| Rajasthan | X |  |  | Low |  |  |
| Sikkim | X | X |  | Intermediate | Sikkim State | Sikkim state |
| Tamil Nadu + Puducherry | X | X | X | High | Chennai, *Dindigul Ambilikkai* | Chennai |
| Uttar Pradesh | X |  |  | Low |  |  |
| Uttarakhand | X |  |  | Low |  |  |
| West Bengal + Andaman & Nicobar Islands | X | X | X | High |  | *Kolkata* |

* States or groups of states as reported in the 2006 National Behavior Surveillance Survey of the National AIDS Control Organization of India.

§ Other North Eastern States include Arunachal Pradesh, Nagaland, Meghalaya, Mizoram, and Tripura.

± States with age-specific cervical cancer incidence data from volume XI of Cancer Incidence in Five Continents (CI5) and the 2012-2016 report of the National Centre for Disease Informatics and Research (NCDIR).

† Type- and age-specific HPV prevalence data.

¤ The eighteen registries CI5 and NCDIR do not have in common are in *italics*.
