# Supplementary material for: Approximating missing epidemiological data for cervical cancer through Footprinting: A case study in India
Source: eLife. 2023 May 25;12:e81752. doi: 10.7554/eLife.81752 (PMC10212556; doi:10.7554/eLife.81752)
Supplement: Figure 2—source data 2. [file elife-81752-fig2-data2.docx]

**Figure 2 – Source Data 2.** **Estimated model parameters under Poisson regression models.**

| **Number of prefixed clusters** | **Cluster label** $\boldsymbol{i}$ | $\boldsymbol{\gamma}_{\boldsymbol{i, intercept}}$ | $\boldsymbol{\gamma}_{\boldsymbol{i, age}}$ | $\boldsymbol{\gamma}_{\boldsymbol{i, age}\boldsymbol{2}}$ |
| --- | --- | --- | --- | --- |
| 2 | 1 | -14.70 | 1.43 | -0.073 |
|  | 2 | -13.64 | 1.50 | -0.085 |
| 3 | 1 | -14.71 | 1.39 | -0.071 |
|  | 2 | -13.66 | 1.52 | -0.086 |
|  | 3 | -14.65 | 1.48 | -0.075 |
| 4 | 1 | -14.66 | 1.39 | -0.071 |
|  | 2 | -13.66 | 1.52 | -0.086 |
|  | 3 | -14.65 | 1.48 | -0.075 |
|  | 4 | -16.16 | 1.62 | -0.086 |

The definition of $\gamma_{i, *}$ is given Appendix S1.
