# Supplementary material for: Approximating missing epidemiological data for cervical cancer through Footprinting: A case study in India
Source: eLife. 2023 May 25;12:e81752. doi: 10.7554/eLife.81752 (PMC10212556; doi:10.7554/eLife.81752)
Supplement: Figure 3—source data 2. [file elife-81752-fig3-data2.docx]

**Figure 3 – Source Data 2. Predictive values of the sexual behavior variables for cervical cancer incidence cluster.**

| **Sexual behavior variable** | **Predictive value** | **Ranking (from high to low predictive value)** |
| --- | --- | --- |
| age.first.sex.Urban.M | -0.0042 | 7 |
| age.first.sex.Urban.F | -0.0031 | 3 |
| age.first.sex.Rural.M | 0.0001 | 2 |
| age.first.sex.Rural.F | -0.0044 | 9 |
| perc.non.regular.Urban.M | 0.0220 | 1 |
| perc.non.regular.Urban.F | -0.0045 | 10 |
| perc.non.regular.Rural.M | -0.0042 | 8 |
| perc.non.regular.Rural.F | -0.0039 | 6 |
| perc.commericial.Urban | -0.0038 | 5 |
| perc.commericial.Rural | -0.0051 | 11 |
| number.commericial.1 | -0.0033 | 4 |
| number.commericial.>3 | -0.0058 | 12 |

Predictive value is defined as the mean decrease in accuracy, which expresses how much the accuracy of the model would decrease if the variable were to be excluded. Higher values correspond to higher predictive values.
