# Supplementary material for: Approximating missing epidemiological data for cervical cancer through Footprinting: A case study in India
Source: eLife. 2023 May 25;12:e81752. doi: 10.7554/eLife.81752 (PMC10212556; doi:10.7554/eLife.81752)
Supplement: Supplementary file 1. [file elife-81752-supp1.docx]

**Appendix** **1.**

**Poisson-regression-based CEM clustering algorithm**

The CEM clustering algorithm employed to cluster registry-specific cervical cancer incidence data, which are count data. The method relies on an iterative process to obtain an optimal clustering based on likelihood under a Poisson regression model. The algorithm operates with a prefixed number of clusters *k*. The iterative process is initialized by an initial C-step and an initial M-step:

- Initial C-step: randomly assign the age-specific cervical cancer incidence $Y_{r}$of each registry *r* to one of the *k* clusters.
- Initial M-step: estimate the initial parameters $\theta^{0}=(\beta_{i, intercept}^{0} , \beta_{i, age}^{0} ,\beta_{i, age2}^{0} )$ under the Poisson regression model as defined in Box 1 based on the initial assignment and compute the proportion of registries $\pi_{c}^{0}$ belonging to each cluster *c*.

Upon initialization, the *m-*th iteration consists of an E-step, C-step, and M-step:

- E-step: compute the probability for the age-specific cervical cancer incidence $Y_{r}$ of each registry $r$ to belong to each cluster *c* given the previously obtained parameter estimates $\theta^{m-1}$:

$$p_{r,c}^{m}=\frac{\pi_{c}^{m-1}f(Y_{r},\theta_{c}^{m-1})}{\sum_{c'}^{k} \pi_{c'}^{m-1}f(Y_{r},\theta_{c'}^{m-1})}.$$

- C-step: assign each the age-specific cervical cancer incidence of each registry to the cluster with the maximum probability.
- M-step: estimate the parameters $\theta^{m}=(\beta_{i, intercept}^{m} , \beta_{i, age}^{m} ,\beta_{i, age2}^{m} )$ under the Poisson regression model as defined under “Poisson regression model” based on the assignment obtained in the C-step and compute the proportion of registries $\pi_{c}^{m}$ belonging to each cluster *c*.

The iterative process terminates when the model fit no longer improves, which is defined as when the difference between two consecutive log-likelihoods is smaller than the given threshold value (0.001).

As different initial assignments could result in different final assignments, the above iterative process was repeated 100 times with different initial assignments, randomly generated from a multinomial distribution. The 100 different final assignments were compared based on the Bayesian information criterion (BIC), and only the one with the highest BIC was selected.

**Poisson regression model**

A Poisson regression model with cluster-specific effects for intercept, age group id $j$($=1,\ldots,13$for 5-year age groups 15-19, …, 75-79) and the square of age group id $j^{2}$ is fitted to the cervical cancer incidence data ($Y$and $t$ for the number of cases and women-years in the data) estimated based on the classified cluster obtained:

$\log\left( E\left( Y \right|j)/t \right)= \left\{ \begin{aligned} \beta_{1, intercept} + \beta_{1,age} \cdot j+\beta_{1,age2} \cdot j^{2}, for cluster i=1, \\ (\beta_{1, intercept}+\beta_{i,intercept})+\left( \beta_{1,age}+\beta_{i,age} \right)\cdot j+{(\beta}_{1,age2}+\beta_{i,age2})\cdot j^{2}, for cluster i>1. \end{aligned} \right.$

For notational convenience, we introduced the following auxiliary coefficients for $*=intercept, age, age2$:

$$\gamma_{i, *} = \left\{ \begin{aligned} \beta_{1, *} , for cluster i=1, \\ \beta_{1, *}+\beta_{i,*} , for cluster i>1. \end{aligned} \right.$$

To facilitate interpretation of the cluster patterns, we then computed the maximum incidence $y_{i,max}$ and the 5-year age group of maximum incidences $j_{i,max}$, as follows:

$y_{i,max} =\exp\left( \gamma_{i, intercept} -\frac{{\gamma_{i, age}}^{2}}{4\cdot\gamma_{i, age2}} \right)\cdot100000$, and $j_{i,max} =\left| \frac{-\gamma_{i, age}}{2\cdot\gamma_{i, age2}} \right|$, $for cluster i$.
